# Treatment of a Case of Cerebro-Spinal Meningitis

**Published:** 1885-06-20

**Authors:** J. McF. Gaston

**Affiliations:** Professor of Principles and Practice of Surgery in the Southern Medical College, Atlanta, Georgia


### TREATMENT OF A CASE OF CEREBRO-SPINAL MENINGITIS.

By J. McF. GASTON, M. D.,
Professor of Principles and Practice of Surgery in the SOUTHERN MEDICAL COLLEGE, Atlanta, Georgia.

[Conclusion of article which appeared in June No. under this caption.]

May 26th, 1885 —As the closing notes of this case failed to appear in the report, it may interest the reader to know that convalescence has progressed slowly, and somewhat irregulariy, with the little patient. My professional attention has been required to his condition on several occasions; at one time on account of disturbance of the bowels, which, being relieved, was followed by gastric trouble, necessitating the use of nourishment and tonics by enemata.

There has been no indication recently of febrile excitement, but, at times, considerable mucous irritability, without any mental disturbance other than fretfulness. He is still feeble, but without any defect of sight or hearing. He has but little relish for food of any kind, but has kept up the milk punch, with soft-boiled eggs and other fluid nourishment, so as to prevent any great emaciation or marked impairment of strength. While confined to the bed, he was using a small branch of a cherry tree, with the leaves on it, in his own hand, to keep off the flies, at my last visit on yesterday.

This result demonstrates the thorough impairment of all the vital forces from this disease, even when arrested promptly, as this was, by the energetic measures resorted to at the outset.

